# Redefining Knee Arthroplasty: Does Robotic Assistance Improve Outcomes Beyond Alignment? An Evidence-Based Umbrella Review

**DOI:** 10.3390/jcm14082588

**Published:** 2025-04-09

**Authors:** Fernando García-Sanz, María Dolores Sosa-Reina, Gonzalo Jaén-Crespo, Ángel González-de-la-Flor, Jorge Hugo Villafañe, Carlos Romero-Morales

**Affiliations:** 1 Faculty of Medicine, Health and Sports, Universidad Europea de Madrid, 28670 Villaviciosa de Odón, Spain; fernando.garcia@clinicacemtro.com (F.G.-S.); mariadolores.sosa@universidadeuropea.es (M.D.S.-R.); gonzalo.jaen@universidadeuropea.es (G.J.-C.); angel.gonzalez@universidadeuropea.es (Á.G.-d.-l.-F.); mail@villafane.it (J.H.V.); 2 Clínica CEMTRO, 28035 Madrid, Spain

**Keywords:** total knee arthroplasty, surgical accuracy, robotic-assisted surgery

## Abstract

**Background**: Robotic-assisted total knee arthroplasty (rTKA) has been introduced to improve surgical precision and alignment in knee replacement procedures. However, its impact on clinical outcomes, pain relief, and cost-effectiveness remains debated. This umbrella review synthesizes evidence from systematic reviews and meta-analyses comparing rTKA to conventional TKA. **Methods**: An umbrella review was conducted in PubMed, Scopus, Web of Science, Embase, and the Cochrane Database of Systematic Reviews. Systematic reviews and meta-analyses comparing rTKA with conventional TKA were included. Methodological quality was assessed using AMSTAR 2 and ROBIS tools. Primary outcomes included hospital stay, radiographic alignment, postoperative pain, functional outcomes, and patient satisfaction. **Results**: Ten systematic reviews were included. rTKA demonstrated superior alignment accuracy and a reduction in alignment outliers. Some studies reported shorter hospital stays and lower early postoperative pain scores for rTKA. However, these benefits did not consistently translate into improved long-term functional outcomes, patient satisfaction, or reduced revision rates. Cost-effectiveness analyses indicated that rTKA remains an expensive option, with benefits largely dependent on surgical volume and healthcare system resources. **Conclusions**: While rTKA improves surgical precision and may offer short-term advantages, its long-term superiority over conventional TKA remains unproven. Higher costs and longer operative times limit its widespread adoption. Further high-quality, long-term studies are needed to determine its clinical and economic value.

## 1. Introduction

Total knee arthroplasty (TKA) is a successful surgical procedure for knee osteoarthritis, with substantial pain relief and functional recovery for most patients [1]. However, despite advances in surgical technique, approximately 15–20% of TKA patients remain poorly satisfied with their outcomes, experiencing pain or limited function [2]. One factor for suboptimal results may be imprecise implant positioning and limb malalignment, which can lead to instability or early wear [3]. In response, computer-assisted surgeries have been introduced with the objective of improving surgical precision [4]. Robotic assistance in TKA has grown in recent years as surgeons and hospitals seek to leverage these technologies to improve patient outcomes [5]. Alignment in TKA refers to the positioning of femoral and tibial components in relation to the mechanical axis of the lower limb. Neutral mechanical alignment is traditionally defined as a hip–knee–ankle (HKA) angle within ±3° of neutral in the coronal plane. Malalignment, typically defined as a deviation greater than 3°, has been associated with increased polyethylene wear, abnormal joint loading, reduced implant survival, and higher revision rates [6].

Several studies have reported that robotic-assisted (rTKA) can reduce alignment outliers and improve the accuracy of component placement, which may translate into faster early recovery, less postoperative pain, and shorter hospital stays [6]. On the other hand, results from meta-analyses have found no significant differences between robotic and traditional TKA in terms of postoperative pain, functional scores, or complication rates [7]. Indeed, some recent systematic reviews and clinical guidelines conclude that aside from improved operative precision, rTKA may offer no clear advantage in patient-reported outcomes or overall success when compared to conventional techniques in the short term [8]. There is conflicting evidence in the orthopedic community regarding the clinical value of robotic assistance in TKA.

Although multiple systematic reviews and meta-analyses have investigated outcomes of robotic-assisted versus conventional TKA, they vary substantially in scope, quality, and outcomes assessed, often reporting conflicting results regarding pain, function, and clinical relevance of alignment improvements. Moreover, these reviews differ in their methodological rigor and frequently fail to evaluate the risk of bias or overall quality using standardized tools. To date, no umbrella review has critically synthesized and appraised this body of literature with a focus on core clinical outcomes. Therefore, the aim of this umbrella review is to consolidate the available evidence, assess the methodological quality of existing reviews, and provide an integrated summary of whether robotic-assisted TKA leads to demonstrable improvements in hospital length of stay, alignment precision, postoperative pain, and functional outcomes compared to conventional TKA [9]. We focus on hospital length of stay, radiographic alignment, postoperative pain, and functional outcomes measured by the Knee injury and Osteoarthritis Outcome Score (KOOS) or Western Ontario and McMaster Universities Osteoarthritis Index (WOMAC) to determine whether rTKA demonstrably improves these parameters compared to conventional TKA [10].

## 2. Materials and Methods

### 2.1. Structure of the Umbrella Review

A general review systematically evaluates and compiles evidence from multiple systematic reviews regarding all outcomes for which they have been conducted. The steps described in the Cochrane Handbook and other methodological documents on conducting umbrella reviews were followed to develop this general review. A protocol was established before conducting this review, developing a comprehensive search strategy to include all systematic reviews and meta-analyses comparing the effects of rTKA and conventional TKA on hospital stay and postoperative physical outcomes, including range of motion, muscle strength, pain, and functional performance. Systematic reviews of both conventional and rTKA were included because both procedures are increasing elective interventions, providing durable joints that effectively relieve pain and restore function in patients with end-stage osteoarthritis. Study selection, data extraction, and the quality assessment of systematic reviews were conducted in duplicate. The validated AMSTAR 2 and ROBIS tools were used to evaluate the methodological quality of the included systematic reviews. The review followed PRISMA guidelines. As this is an umbrella review, there is no registration number.

### 2.2. Search Strategy

Searches were conducted in PubMed, Scopus, Web of Science, Embase, and the Cochrane Database of Systematic Reviews to identify systematic reviews published up to January 2025 that compared conventional and rTKA. The search strategy combined various terms adapted to each database to collect the maximum number of reviews. For example, for PubMed: (“Robotic-assisted surgery” OR “Robot-assisted total knee arthroplasty” OR “Robotic TKA”) AND (“Total knee arthroplasty” OR “Knee replacement surgery”) AND (“Hospital stay” OR “Length of stay”) AND (“Physical function” OR “Range of motion” OR “Muscle strength” OR “Post-operative outcomes”).

### 2.3. Selection and Screening of Systematic Reviews

This process was conducted in two stages. The first stage involved selecting titles and abstracts based on inclusion and exclusion criteria. The second stage focused on the full-text selection of studies that met the eligibility criteria. Two independent reviewers conducted this process, resolving any disagreements through discussion or consultation with a third reviewer.

A standardized data extraction form was used to collect the following information from each included systematic review or meta-analysis:

- Study characteristics (author, year, population, sample size, type of robot-assisted system).
- Outcomes of interest (hospital stay, physical outcomes).
- Risk of bias assessment (as reported in the review or meta-analysis).
- Effect sizes (e.g., mean difference, standardized mean difference) for hospital stay and physical outcomes.

Systematic reviews were collected as defined by the Preferred Reporting Items for Systematic Reviews and Meta-Analyses (PRISMA) statement. The included reviews met the following inclusion criteria: systematic reviews and meta-analyses comparing robot-assisted and conventional TKA, studies reporting outcomes related to hospital stay and physical function, and studies published in English. The included reviews evaluated various robotic systems, including image-based (e.g., MAKO), image-free (e.g., ROBODOC), and semi-active systems (e.g., CASPAR). However, technical differences exist between these platforms, such as preoperative planning, haptic feedback, and registration techniques. Our inclusion criteria accepted any robotic-assisted TKA system as long as it was compared to conventional TKA and reported data on at least one of the primary outcomes (hospital stay, alignment, pain, function). Regarding exclusion criteria, reviews focusing on TKA for conditions other than knee osteoarthritis (e.g., fractures), reviews where robotic and conventional TKA were not the primary interventions of interest, and studies with insufficient data on primary outcomes (hospital stay or physical function) were excluded. Two reviewers (GJ and MDS) independently extracted data from the included systematic reviews. Extracted data included general information about the systematic review (e.g., year of publication, journal, and funding sources) and details on interventions, study design, and key findings of the studies included in the reviews.

### 2.4. Methodological Quality Assessment

After agreeing on study inclusion, three reviewers (GJ, MDS) independently assessed the methodological quality of the included reviews using the “A Measurement Tool to Assess Systematic Reviews 2” (AMSTAR 2) and ROBIS. In case of disagreement, a consensus was reached through the intervention of a third reviewer (AGF).

First developed in 2007 (as AMSTAR) to assess the methodological quality of systematic reviews synthesizing evidence from randomized trials, this assessment tool was further developed as AMSTAR 2 in 2017 to expand its use to systematic reviews of both randomized and non-randomized studies [11]. AMSTAR 2 comprises 16 domains, seven of which are critical domains as they significantly undermine confidence in the conclusions of systematic reviews: one domain relates to protocol registration, two relate to search strategy (adequacy and justification for study exclusion), two relate to the risk of bias assessment of included studies and its effect on systematic review conclusions, one relates to evidence synthesis methodology, and one relates to publication bias. Overall confidence in systematic review results is classified into four categories: high (no or one non-critical weakness), moderate (more than one non-critical weakness), low (one critical flaw with or without non-critical weaknesses), and very low (more than one critical flaw with or without non-critical weaknesses). Therefore, AMSTAR 2 has been considered a valid and reliable instrument, like other systematic review assessment tools [11].

Moreover, ROBIS is currently aimed at four broad categories of reviews, primarily within healthcare settings: interventions, diagnosis, prognosis, and etiology. The target audience for ROBIS includes guideline developers, authors of systematic review summaries (“reviews of reviews”), and reviewers who may want to assess or avoid bias in their reviews. The tool is completed in three phases: (1) assessing relevance (optional), (2) identifying concerns with the review process, and (3) judging the risk of bias. Phase 2 covers four domains through which bias may be introduced into a systematic review: study eligibility criteria, study identification and selection, data collection and study assessment, and synthesis and findings. Phase 3 evaluates the overall risk of bias in interpreting review findings and whether it considered the limitations identified in any of the Phase 2 domains. Signaling questions are included to help assess concerns with the review process (Phase 2) and the overall risk of bias in the review (Phase 3); these questions highlight aspects of review design related to the potential for bias and aim to help assessors judge the risk of bias in the review process, results, and conclusions. ROBIS is the first rigorously developed tool specifically designed to assess the risk of bias in systematic reviews and is, therefore, considered essential in evaluating the methodological quality of any review [12].

## 3. Results

**Figure 1:**
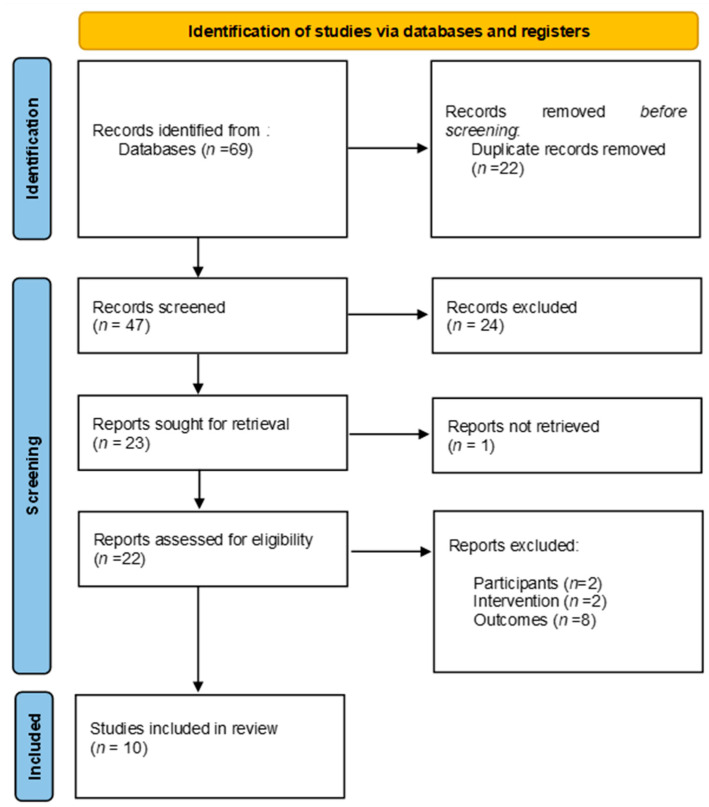
Flow diagram of study selection process.

Following the removal of duplicates, a total of 69 studies were identified. After screening titles and abstracts, 24 studies were excluded for not meeting the predefined inclusion criteria. Of the remaining studies, one was excluded due to the unavailability of the full text, and 12 were excluded as they did not align with the inclusion criteria regarding the population, interventions, or outcomes of interest. Consequently, 10 systematic reviews were included (Figure 1).

### 3.1. Description of the Included Systematic Reviews

The included studies were published between 2013 and 2024, with most appearing after 2020. These studies compare rTKA with conventional manual or computer-assisted techniques, including evaluations of specific robotic platforms such as MAKO, ROBODOC, and CASPAR. Sample sizes varied significantly, ranging from 402 participants in the study by Mannan et al. [13] to 9084 participants in the study by Batailler et al. [14], with distinct groups for robotic and conventional TKA approaches. A wide range of outcomes was assessed, including hospital stay duration and physical variables such as joint range of motion, pain, and functionality (Table 1).

### 3.2. Methodological Quality of the Included Systematic Reviews

**Figure 2:**
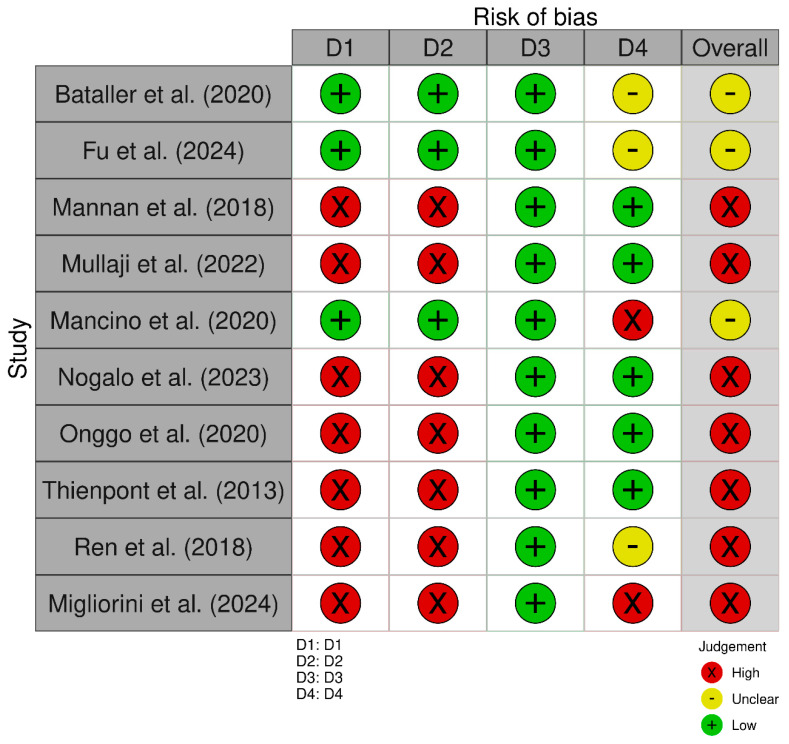
Results of the risk of bias assessment for included systematic reviews [13,14,15,16,17,18,19,20,21,22].

According to AMSTAR 2, confidence in the results of 4 studies was rated critically low [14,16,20,21], 1 study was rated low [17], 2 studies were rated moderate [13,19], and 3 studies were rated high [15,18,22]. Critically low confidence was attributed to factors such as the lack of a developed protocol (reported in 2 out of the 4 critically low studies) [16,21], failure to assess the risk of bias in the included studies (4/4) [14,16,20,21], and failure to account for the risk of bias when interpreting the results (4/4) [14,16,20,21]. Additionally, low and moderate confidence ratings were associated with partial reporting of excluded studies and inadequate justification for exclusions. Figure 2 illustrates the prevalence of critical flaws and non-critical weaknesses across the included systematic reviews. Table 2 provides a detailed assessment of weaknesses for each study.

### 3.3. Risk of Bias in the Included Systematic Reviews

According to the ROBIS assessment, overall concerns were high in 7 studies [1,3,6,9,10] and unclear in 3 studies [14,15,16]. The main reasons behind the high overall concerns are related to domains D1 (Study eligibility criteria) and D2 (Identification and selection of studies). In the 7 studies [13,15,18,21,22], difficulties were observed in the study selection process, with unclear or insufficiently justified inclusion and exclusion criteria (D1), as well as issues with the identification and selection of relevant studies (D2), which may have affected the quality of the included studies.

Additionally, some studies [13,15,18,21,22] did not adequately meet the requirements of domain D3 (data collection and study appraisal). This domain includes aspects such as insufficient detail in the data extraction process, inconsistencies in the validation of the collected data, and omissions in the assessment of risk of bias, which contributed to greater concerns about the quality and reliability of the results.

In domain D4 (Synthesis and findings), weaknesses were also found in how the results were synthesized and reported [14,16], which may have influenced the interpretation and presentation of the evidence (Figure 2).

### 3.4. Summary and Consistency of the Evidence

#### 3.4.1. Hospital Stay

The length of hospital stay after rTKA has been shown to be shorter compared to conventional TKA. Batailler et al. [14] reported an average stay of 77 h in patients undergoing rTKA, significantly lower than the 105 h observed in conventional TKA. Similarly, Mullaji et al. [21] indicated that in 4 out of 6 studies analyzed, hospital stays were shorter in the rTKA group, ranging between 0.48 and 2.1 days. These findings consistently indicate that hospital stay is generally shorter for patients undergoing robot-assisted total knee arthroplasty compared to conventional manual techniques.

#### 3.4.2. Postoperative Alignment

Robotic systems have demonstrated improved mechanical alignment precision in TKA. In the study by Ren et al. [22], the rate of outliers (>3° deviation) in the coronal and sagittal alignment of the femur and tibia was significantly lower in the rTKA group. Thienpont et al. [20] reported a reduction in the probability of misalignment greater than 2° and 3° in surgeries assisted by computerized navigation. Fu et al. [15] highlighted that the hip–knee–ankle (HKA) angle alignment was more precise and consistent in rTKA, although these benefits did not always translate into better long-term clinical outcomes. These data indicate that robotic-assisted total knee arthroplasty achieves greater alignment accuracy compared to conventional methods, with fewer deviations from optimal alignment angles.

#### 3.4.3. Range of Motion

Findings on postoperative range of motion are contradictory. Batailler et al. [14] reported that the average flexion at discharge was higher in the rTKA group (104.1°) compared to conventional TKA (93.3°), with sustained improvement at 90 days. However, Fu et al. [15] found that in the long term (>6 months), the range of motion was significantly lower in the rTKA group (weighted mean difference of −2.84°, *p* = 0.00001). Therefore, although rTKA provides greater flexion in the short term, studies show that, in the long term, the range of motion is comparable to or even inferior to that of conventional TKA.

#### 3.4.4. Postoperative Pain

Studies demonstrate a reduction in immediate postoperative pain with rTKA. Batailler et al. [14] reported lower pain scores on the visual analog scale (3.6 vs. 6.3) during hospitalization in rTKA patients. However, Fu et al. [15] indicated that long-term pain outcomes do not significantly favor rTKA over conventional TKA.

#### 3.4.5. Functional Performance

Postoperative functional evaluations show initial advantages for rTKA, although these benefits may decrease over time. Batailler et al. [14] reported that rTKA patients achieved better scores in WOMAC (6 ± 6 vs. 9 ± 8) and in functional KSS (80 vs. 73) at one year postoperatively. However, Fu et al. [15] highlighted that improvement in these scores was lower at >6 months of follow-up compared to conventional TKA. These findings suggest that although robotic technology enhances surgical precision, its impact on long-term functionality remains uncertain.

## 4. Discussion

The present umbrella review synthesized evidence from systematic reviews and meta-analyses comparing rTKA with conventional TKA. Our findings suggest that while robotic systems improve implant alignment and reduce certain postoperative complications, their clinical benefits in terms of long-term functional outcomes and pain relief remain uncertain. These conclusions align with prior reviews in orthopedic surgery, which have often found technological innovations to enhance procedural accuracy without necessarily translating into superior patient-reported outcomes [22]. 

One of the most consistent findings across the included systematic reviews was the improved radiographic alignment with rTKA compared to conventional techniques. Several studies demonstrated that robotic assistance reduces the risk of alignment outliers, potentially leading to better implant longevity [12,23]. A study by Kaneko et al. found that rTKA reduced outliers in rotational alignment of the tibial prosthesis compared to conventional TKA, which can enhance the alignment and congruency of the femoral-tibial surfaces [24]. Similarly, a study conducted by Song E. et al. reported that rTKA decreased the number of the mechanical axis alignment outliers and improved the ability to achieve flexion-extension gap balance without differences in clinical scores with respect to the conventional approach for TKA [25]. Improved alignment is theorized to contribute to reduced polyethylene wear and lower revision rates in the long term; however, no high-quality evidence currently confirms this assumption. The findings provided by Fu et al. suggest that while alignment accuracy is enhanced, this has not been correlated with significant reductions in revision rates or improvements in long-term functional outcomes [13]. However, several included reviews reported that rTKA improves coronal alignment precision and reduces mechanical axis outliers (>3°), a threshold commonly used to define malalignment. However, these studies did not examine whether such improvements are associated with lower revision rates or improved long-term outcomes. Moreover, differences in alignment strategies (e.g., mechanical, kinematic, or gap-balancing approaches) were not consistently reported across reviews, making it difficult to interpret the clinical relevance of alignment deviations.

Although rTKA improves alignment accuracy, this does not consistently lead to better functional outcomes. This discrepancy may be due to the multifactorial nature of recovery, where factors such as soft tissue balance, patient expectations, preoperative status, and rehabilitation adherence play a major role. Moreover, radiographic alignment does not always correlate with kinematic function or patient satisfaction. Standard outcome measures may also lack sensitivity to detect clinically meaningful differences related solely to alignment.

Regarding the hospital stay, several reviews indicated that rTKA might contribute to shorter postoperative hospitalization. This may be due to reduced soft tissue trauma, improved surgical precision, and better early recovery approaches [12,19]. For instance, Maman et al. found that rTKA patients had a shorter length of stay compared to those undergoing navigation-guided techniques [26]. Similarly, Kayani et al. reported a median time to hospital discharge of 77 h for rTKA patients, compared to 105 h for conventional jig-based TKA [27].

However, the cost-effectiveness of this potential advantage remains a topic of debate, as robotic surgery incurs significantly higher initial and maintenance costs, which could limit its widespread adoption [16]. For instance, a cost-effectiveness analysis indicated that rTKA was cost-effective only in high-volume hospitals performing over 49 procedures annually, with an incremental cost-effectiveness ratio of $41,331 per quality-adjusted life-year (QALY) [28]. Similarly, another study found that rTKA was not a cost-effective alternative to conventional TKA, with an incremental cost of $128,526 per QALY [29]. Despite shorter hospital stays, rTKA is associated with longer operative times, potentially increasing surgical workload and resourced utilization in high-volume facilities.

In terms of postoperative pain and functional recovery, the evidence is mixed. Some systematic reviews suggest an initial advantage for rTKA in reducing early postoperative pain, possibly due to more precise bone cuts and ligament balancing [13,14]. Enhanced soft tissue preservation may contribute to lower inflammatory responses, leading to reduced pain in the immediate postoperative stage. Batailler et al. reported that patients undergoing rTKA had lower early pain scores on the VAS scale compared with those undergoing conventional TKA [12]. In this line, Kayani et al. observed that patients undergoing rTKA reduced postoperative pain intensity, decreased analgesia requirement, and decreased the time to reach a proper straight leg raise compared with those with conventional TKA [27]. However, this benefit did not persist at later follow-up, with no significant differences in pain levels beyond the six-month follow-up.

Considering functional outcomes, several authors support that robotic assistance allows for better ligament balancing, potentially optimizing knee kinematics and stability in the early postoperative period. For instance, recent research showed that functional alignment achieved higher balance scores in both extension and flexion following the rTKA, leading to significant clinical outcomes at the two-year follow-up [30]. However, long-term functional advantages remain unclear. Fu et al. argued that while some patients exhibited improved functional scores within the first three months postoperatively, these decreased over time, converging with outcomes observed in conventional TKA groups [13]. Although some reviews reported statistically significant differences in pain and function favoring rTKA, these improvements often fell below the minimal clinically important difference (MCID) thresholds established for commonly used outcome measures. For example, the MCID for WOMAC ranges from 9 to 12 points, and for KOOS subscales, from 8 to 10 points. In the included reviews, most reported differences in these scores were smaller, suggesting that the clinical relevance of such improvements is limited. Moreover, Ren et al. reported that while rTKA patients demonstrated slightly improved ROM at the postoperative stage, this did not translate into significantly better patient satisfaction or daily activity scores compared with conventional TKA groups [20]. Similarly, a multicenter cohort study reported that patients undergoing rTKA experienced immediate postoperative gains in ROM; these advantages were considered clinically modest, and additional research is still necessary [31].

Patient satisfaction is a critical measure of success following TKA. While improved radiographic alignment with rTKA may theoretically enhance outcomes, patient-reported measures remain the gold standard for assessing surgical success [32]. Studies have reported mixed findings regarding patient satisfaction with rTKA with respect to conventional TKA. For instance, a systematic review with meta-analysis found no statistically significant difference in satisfaction rates between rTKA and conventional TKA, with satisfaction rates of 95% and 91%, respectively [33]. Several factors may contribute to these discrepancies in patient satisfaction, including preoperative expectations, rehabilitation adherence, and individual biomechanical variations. Unmet expectations have been identified as a strong predictor of dissatisfaction after TKA [32]. These findings highlighted the multifactorial nature of patient satisfaction and the importance of addressing both clinical and individual factors to optimize outcomes following TKA.

Additionally, rTKA has been associated with certain complications not observed in conventional procedures. A comprehensive review identified specific issues such as pinhole fractures, related infections, iatrogenic soft tissue and bone injuries, and excessive blood loss associated with robotic systems [15,16,17,18]. Additionally, Pagani et al. analyzed adverse events reported to the U.S. Food and Drug Administration and highlighted incidents like unexpected robotic arm movements, retained foreign objects, and surgical delays, some requiring conversion to manual surgeries. While overall complication rates between rTKA and conventional TKA are comparable, these risks should be considered when evaluating the widespread implementation of robotic systems [34].

Despite its benefits, robotic-assisted TKA presents specific drawbacks. These include longer operative times, which may raise infection risk, and complications related to tracking pins, such as pin-site infections or fractures. Additionally, robotic systems require a learning curve, and early use may increase the risk of soft-tissue or alignment errors. Although overall complication rates are comparable to conventional TKA, the nature of complications is unique and should be considered in clinical decision-making.

## 5. Limitations

This umbrella review has several limitations that must be acknowledged. First, the heterogeneity among the included systematic reviews and meta-analyses is a significant concern. Variations in study design, patient populations, surgical techniques, robotic platforms, and outcome measures make direct comparisons challenging. Another source of heterogeneity is the diversity of robotic platforms included across studies. Differences in image-based vs. image-free navigation, haptic guidance, and system learning curves may have influenced outcomes, such as alignment accuracy, operative time, or early recovery, but these aspects were not consistently reported across reviews. According to the AMSTAR 2 assessment, several studies were rated as critically low due to inadequate risk-of-bias assessment, incomplete protocol descriptions, and lack of a predefined systematic approach to evidence synthesis. The ROBIS evaluation further highlighted concerns related to study selection criteria, data extraction inconsistencies, and the interpretation of results, which may impact the reliability of the synthesized findings. Second, most included studies focused on short-term and intermediate-term outcomes, such as early pain relief, radiographic alignment, and initial functional recovery. However, key long-term variables, including implant survival, revision rates, and patient satisfaction beyond five years, were insufficiently addressed. Given that TKA is a long-term intervention, future research should prioritize extended follow-up periods to assess whether the benefits of robotic surgery translate into improved long-term durability. Finally, publication bias may have influenced the results of this review. Studies with positive findings favoring rTKA are more likely to be published than those with neutral or negative results. This bias may have led to an overestimation of the benefits of robotic systems while underreporting potential drawbacks. Future studies should include prospective registry-based analyses and randomized controlled trials with predefined primary outcomes to provide a more balanced and comprehensive evaluation.

## 6. Future Directions

Future research should focus on long-term, high-quality randomized controlled trials comparing rTKA and conventional TKA with follow-up periods beyond five years, focusing on implant survival, revision rates, and sustained patient-reported outcomes. Studies should also explore subgroup effects, identifying patients who may benefit most from robotic assistance (e.g., those with severe deformity, obesity, or high activity levels). Large-scale registry-based analyses can complement trial data by capturing real-world outcomes and complications. Furthermore, detailed cost-effectiveness analyses are needed across various healthcare systems to determine under what conditions robotic surgery provides economic value. Finally, future trials should ensure that outcome measures include not only radiographic and surgical metrics but also MCID-based patient-centered outcomes, such as pain, function, and satisfaction.

## 7. Conclusions

Robotic-assisted TKA improves alignment accuracy and may reduce hospital stay compared to conventional techniques. However, current evidence does not support consistent advantages in long-term functional outcomes, pain relief, or revision rates. Although alignment precision is enhanced, these improvements often fall below clinically meaningful thresholds and do not reliably translate into better patient-reported outcomes. Given the higher costs and longer operative times associated with rTKA, its widespread adoption should be considered cautiously and guided by further high-quality, long-term, and cost-effectiveness research.

## Figures and Tables

**Table 1:** Characteristics of included systematic reviews.

Study	Included Studies	Participants	Intervention	Comparison	Outcomes	Conclusions/Results
Batailler et al., 2020 [14]	14	9084 (3090 RA-TKAs, 5994 conventional TKA)	MAKO CT-based robotic arm-assisted TKA	Conventional manual TKA	Accuracy of alignment, pain, hospital stay, soft tissue injury	RA-TKA improves alignment accuracy and reduces soft tissue injuries but is costlier and time-intensive.
Fu et al., 2024 [15]	12	2863 (1449 RA-TKAs and 1414 M-TKAs)	Robotic-assisted total knee arthroplasty (RA-TKA)	Manual total knee arthroplasty (M-TKA)	KSS, WOMAC, OKS, ROM, HKA angle, component angles, satisfaction	RA-TKA yields better alignment accuracy but no superior clinical outcomes compared to M-TKA over time.
Mancino et al., 2020 [16]	9	1199 (614 RA, 585 CM)	Robotic (RA) TKA	Conventional (CM) TKA	Implant survivorship, complications, clinical outcomes, radiographic outcomes	Improved radiographic outcomes (fewer radiolucent lines and deviations in RA); no significant differences in operative time and complications.
Mannan et al., 2018 [13]	5	402	Robotic-assisted TKA	Conventional TKA	Mechanical axis alignment, tibial and femoral alignment, operative time, blood loss	Robotic-assisted TKA achieved significantly more accurate alignment with fewer mechanical axis deviations (>3°) compared to conventional TKA. Additionally, reduced blood loss and longer operative times were observed.
Migliorini et al., 2024 [17]	7	1198 (100 conventional TKA, 1098 RA-TKAs or computer-assisted)	Robotic (RA) TKA	Conventional (CM) TKA or computer assisted	Resection accuracy, implant alignment, gap balancing	FA shows better results in precision and alignment, but more long-term evidence is needed.
Nogalo et al., 2023 [18]	21	Not specified	Robotic TKA	Conventional TKA	Complications, operative duration, costs, infection rates	Robotic TKA associated with complications such as pin-hole fractures, iatrogenic injuries, excessive blood loss, and pin-site infections. Longer surgical duration and higher intraoperative costs were also reported.
Onggo et al., 2020 [19]	18	6534 (2234 RA, 4300 CM)	Robotic (rTKA)	Conventional (cTKA)	Clinical outcomes, radiographic outcomes, complications, perioperative parameters, and costs	rTKA reduces blood loss and improves alignment, but the long-term clinical impact is marginal.
Thienpont et al., 2013 [20]	30	Not specified	Computer-assisted navigation, Patient-Matched Instrumentation (PMI), Robotic-assisted implantation	Conventional instrumentation	Mechanical alignment, operative time, malalignment	Computer-assisted navigation reduced the number of alignment deviations (>2° and >3°) compared to conventional instrumentation. Patient-Matched Instrumentation (PMI) and robotic implantation showed mixed results, with some improvements in alignment but limited demonstrated clinical benefits
Mullaji et al., 2022 [21]	13	2112 knees	Robotic-Assisted Total Knee Arthroplasty (RATKA)	Conventional Total Knee Arthroplasty (CTKA)	Operative time, Length of stay, Functional outcomes, Alignment, Complications	Robotic-assisted TKA provides better component alignment and early functional outcomes. However, there is limited evidence of its superiority over CTKA in terms of long-term outcomes. Operative times are generally longer for RATKA, but hospital stays may be shorter. Complication rates are similar between RATKA and CTKA.
Ren et al., 2018 [22]	7 studies (6 RCTs and 1 retrospective)	486 patients, 517 knees	Robot-assisted TKA (ROBODOC, CASPAR)	Conventional TKA	Accuracy of mechanical alignment and implant positioning; Functional scores (WOMAC, KSS, HSS); Complications (blood loss, surgical time, ROM)	Improved mechanical alignment and implant positioning accuracy; Better functional scores (WOMAC, KSS); Reduced blood loss; No significant differences in surgical time, ROM, or complication rates; Further studies are needed.

RA-TKA: Robotic-Assisted Total Knee Arthroplasty; M-TKA: Manual Total Knee Arthroplasty; KSS: Knee Society Score; WOMAC: Western Ontario and McMaster Universities Osteoarthritis Index; OKS: Oxford Knee Score; ROM: Range of Motion; HKA: Hip-Knee-Ankle; RA: Robotic-Assisted; CM: Conventional Manual; CTKA: Conventional Total Knee Arthroplasty; CASPAR: Computer-Assisted Surgery Positioning and Alignment Robot; PMI: Patient-Matched Instrumentation; RATKA: Robotic-Assisted Total Knee Arthroplasty.

**Table 2:** Results of AMSTAR-2.

Study	1	2	3	4	5	6	7	8	9	10	11	12	13	14	15	16	Overall
Batailler et al., 2020 [14]	Yes	Partial Yes	Yes	Yes	No	No	No	Partial Yes	Yes	No	NA	NA	No	Yes	NA	Yes	Critically low
Fu et al., 2024 [15]	Yes	Yes	Yes	Yes	Yes	Yes	No	No	Yes	No	Yes	Yes	Yes	Yes	Yes	Yes	High
Mancino et al., 2020 [16]	Yes	No	Yes	Yes	Yes	Yes	No	Yes	Partial Yes	No	NA	NA	Yes	No	NA	Yes	Critically low
Mannan et al., 2018 [13]	Yes	Partial Yes	Yes	Yes	Yes	Yes	No	Yes	Yes	No	Yes	Yes	Yes	Yes	Yes	Yes	Moderate
Migliorini et al., 2024 [17]	Yes	Yes	Yes	Yes	Yes	Yes	No	Yes	Yes	No	NA	NA	Yes	No	NA	Yes	Low
Mullaji et al., 2022 [21]	Yes	No	Yes	Yes	Yes	No	No	Yes	No	No	NA	NA	No	No	NA	Yes	Critically low
Nogalo et al., 2023 [18]	Yes	Yes	Yes	Yes	Yes	Yes	No	Yes	Yes	No	NA	NA	Yes	Yes	NA	Yes	High
Onggo et al., 2020 [19]	Yes	Partial Yes	Yes	Yes	Yes	Yes	No	Yes	Yes	No	Yes	Yes	Yes	Yes	Yes	Yes	Moderate
Ren et al., 2018 [22]	Yes	Yes	Yes	Yes	Yes	Yes	No	No	Yes	No	Yes	Yes	Yes	Yes	Yes	Yes	High
Thienpont et al., 2013 [20]	Yes	Partial Yes	Yes	Yes	Yes	Yes	No	Yes	No	No	NA	NA	No	No	NA	Yes	Critically low

## Data Availability

Not applicable.
